# Mitochondrial Genome Sequence and Expression Profiling for the Legume Pod Borer *Maruca vitrata* (Lepidoptera: Crambidae)

**DOI:** 10.1371/journal.pone.0016444

**Published:** 2011-02-02

**Authors:** Venu M. Margam, Brad S. Coates, Richard L. Hellmich, Tolulope Agunbiade, Manfredo J. Seufferheld, Weilin Sun, Malick N. Ba, Antoine Sanon, Clementine L. Binso-Dabire, Ibrahim Baoua, Mohammad F. Ishiyaku, Fernando G. Covas, Ramasamy Srinivasan, Joel Armstrong, Larry L. Murdock, Barry R. Pittendrigh

**Affiliations:** 1 Department of Entomology, Purdue University, West Lafayette, Indiana, United States of America; 2 United States Department of Agriculture – Agricultural Research Service, Corn Insect and Crop Genetics Research Unit, Genetics Laboratory, Iowa State University, Ames, Iowa, United States of America; 3 Department of Entomology, University of Illinois at Urbana-Champaign, Champaign, Illinois, United States of America; 4 Department of Crop Sciences, University of Illinois at Urbana-Champaign, Champaign, Illinois, United States of America; 5 Station de Kamboinsé,Institut de l'Environnement et de Recherches Agricoles (INERA), Ouagadougou, Burkina Faso; 6 Institut National de Recherche Agronomique du Niger, Maradi, Niger; 7 Department of Plant Science, Institute for Agricultural Research, Ahmadu Bello University, Zaria, Nigeria; 8 University of Puerto Rico, Mayaguez, Puerto Rico, United States of America; 9 AVRDC-The World Vegetable Center, Tainan, Taiwan; 10 Entomology, The Commonweatlth of Scientific and Industrial Research Organization, Black Mountain, Australian Capital Territory, Australia; Ghent University, Belgium

## Abstract

We report the assembly of the 14,054 bp near complete sequencing of the mitochondrial genome of the legume pod borer (LPB), *Maruca vitrata* (Lepidoptera: Crambidae), which we subsequently used to estimate divergence and relationships within the lepidopteran lineage. The arrangement and orientation of the 13 protein-coding, 2 rRNA, and 19 tRNA genes sequenced was typical of insect mitochondrial DNA sequences described to date. The sequence contained a high A+T content of 80.1% and a bias for the use of codons with A or T nucleotides in the 3rd position. Transcript mapping with midgut and salivary gland ESTs for mitochondrial genome annotation showed that translation from protein-coding genes initiates and terminates at standard mitochondrial codons, except for the *cox*I gene, which may start from an arginine CGA codon. The genomic copy of *cox*II terminates at a T nucleotide, and a proposed polyadenylation mechanism for completion of the TAA stop codon was confirmed by comparisons to EST data. EST contig data further showed that mature *M. vitrata* mitochondrial transcripts are monocistronic, except for bicistronic transcripts for overlapping genes *nd*4/*nd*4L and *nd*6/*cyt*b, and a tricistronic transcript for *atp*8/*atp*6/*cox*III. This processing of polycistronic mitochondrial transcripts adheres to the tRNA punctuated cleavage mechanism, whereby mature transcripts are cleaved only at intervening tRNA gene sequences. In contrast, the tricistronic *atp*8/*atp*6/*cox*III in *Drosophila* is present as separate *atp*8/*atp*6 and *cox*III transcripts despite the lack of an intervening tRNA. Our results indicate that mitochondrial processing mechanisms vary between arthropod species, and that it is crucial to use transcriptional information to obtain full annotation of mitochondrial genomes.

## Introduction

The mitochondrial genome encodes genes involved in oxidative phosphorylation and a unique translation system of 2 rRNAs and 22 tRNAs used for synthesis of the 13 inclusive protein coding genes (PCGs). Within animal species the mitochondrial genome shows a lack of introns and little intergenic space, the exception being the AT nucleotide rich displacement loop (D-loop) which encodes the origin of replication and promoters for the translation of both the heavy (H) and the light (L) strands (heavy strand promoter, HSP; light strand promoter, LSP) [1]. Thus, both H and L strands of the mitochondrial genome are transcriptionally active, and produce polycistronic RNA transcripts (cistrons) [2], [3], [4]. The tRNA regions form characteristic cloverleaf secondary structure within the initial polycistronic transcripts, and they are recognized by cleavage mechanisms that result in processed transcripts that encode one or more PCGs [5]. This mode of gene expression is reminiscent of the bacterial cistronic mode of gene expression from which eukaryotic mitochondria are believed to have been derived. As a consequence of gene order in the mitochondrial genome, the tRNA-punctuated mode of transcript processing can result in mature transcripts that encode more than one PCG [5], where bicistronic transcripts are predicted for mitochondrial PCG sequences (cds) that overlap within the genomic sequence, which includes *atp*8/*atp*6 and *nd*4/*nd*4L within insect mitochondria.

Mitochondrial genomes have been sequenced for insect species within the order Lepidoptera: the first ones sequenced were from the silk moth *Bombyx mori* [6] and the corn borer *Ostrinia spp.* [7]. Lepidopteran mitochondrial genomes have a relatively conserved gene order and orientation [8], which is shared with that of *Drosophila* species [9]. Despite the generation of full mitochondrial genome sequences, the corresponding annotation of gene coding regions in Lepidoptera has largely relied upon comparison to *Drosophila* gene models. There are discrepancies in mitochondrial gene boundaries in Lepidoptera, among them (1) the ambiguity in the translation start site of cytochrome *c* oxidase subunit I (*cox*I) at either a proposed arginine codon (CGR) or a TTAG site [6], and (2) the assumed polyadenylation following a T nucleotide at the terminus of *cox*II that would complete a truncated stop codon [9]. Expressed sequence tags (ESTs) are a source of gene expression information, and are typically inclusive of mitochondrial-derived transcripts. The use of EST data to assemble mitochondrial-derived transcripts has proven valuable in characterization of gene boundaries, polycistronic transcripts, and differential transcript processing among tissues, as well as for the quantification of mitochondrial transcript stability [10],[11]. Despite the utility of ESTs in the annotation of mitochondrial genomes, these data are rarely incorporated into annotation efforts.

In this paper, we describe the nearly complete mitochondrial DNA sequence for the legume pod borer (LPB), *Maruca vitrata* Fabricius (Lepidoptera: Pyraloidea: Crambidae). This insect species is a crop pest found throughout tropical and subtropical regions of the world. Larval stages of LPB feed upon leaves, flowers and pods of leguminous plants [12], [13], [14], [15] and cause significant yield loss to legume crops cultivated in Southeast Asia [16], [17], [18], South Asia [19], [20], [21], and Central America and South Americas [22], [23]. Its most significant impact is in sub-Saharan Africa [24], [25], [26], [27], where between 20–80% yield reductions are incurred [25]. Accordingly, *M. vitrata* has been identified as a major emerging threat to legume production in developing and under-developed nations. The mitochondrial genome information provided here complements the mitochondrial *cox*I DNA markers developed by Margam et al. (2010) [28], by providing additional sequence data to assist in defining population structure, and gene flow, which will hopefully lead to sustainable and economically viable methods of pest management. With the exception of *Drosophila melanogaster*, this study provides the first instance where (i) mitochondrial genome annotations have been validated by transcribed sequence data, and (ii) predictions of processed mitochondrial transcripts (cistron) have been used for both structural and functional annotation of genes. This added value information suggests that available EST information is a valuable resource for mitochondrial genome annotations.

## Materials and Methods

### Sequence and annotation of the *M. vitrata* mitochondrial genome

Genomic DNA was isolated from a *M. vitrata* specimen (from Burkina Faso; BUR38) [28] using a DNeasy animal tissue kit following the manufacturer's instructions (Qiagen, Valencia, CA). A total of 100 ng genomic DNA was used as the template in 50 µl PCR volumes that also contained 10 pmol primers, 5 µl 10× PCR buffer, 0.4 µl *Taq* polymerase (New England Biolabs, Ipswich, MA), and 1.2 µl 10mM dNTP. Polymerase chain reaction (PCR) was carried out in an Eppendorf Mastercycler thermocycler (Eppendorf, Hamburg, Germany) using the thermal cycling profile of 95°C denaturation for 2 min, followed by 35 cycles of 95°C for 30 s, 52°C for 45 s, 72°C for 1 m, as well as a final cycle of 72°C for 8 m. The PCR products were cleaned to remove any residual primers and nucleotides using Qiaquick PCR purification kits (Qiagen, Valencia, CA) following the manufacturer's protocols. The PCR products were cycle sequenced using 1 µl of purified template, 1 µl of 10× BigDye™ (ABI PRISM™ BigDye™ Terminator Cycle Sequencing Ready Reaction Kit; Applied Biosystems, Foster City, CA), 1 pmol forward or reverse primer (Table 1), and 6 µl of ddH_2_O using the conditions: 95°C for 2 min, followed by 98 cycles of 95°C for 10 s, 50°C for 5 s, and 60°C for 4 min. Cycle-sequencing products were precipitated using an ethanol-sodium acetate procedure. Precipitates were dissolved in 35 µl of ddH_2_O. From this solution, 15 µl was separated on an ABI 3500 Sequencer (Applied Biosystems, Foster City, CA). Sequences from PCR fragments obtained from BUR38 and PR08 were assembled independently using the NextGENe software (Softgenetics®, State College, PA), and the assemblies were exported in FASTA format.

**Table 1:** Oligonucleotide primers used for amplification of overlapping fragments used to reconstruct the *M. vitrata* mitochondrial genome sequence (Fig. 2).

Primer pair	Forward	Reverse
ND2	TCTTGATTTGGWTGYTGAATTGG	GTWCCAATATCTTTATGATTTGTTGAG
COI	TCGCTTATAACGTCAGCCATTT	CTAATCAATTTCCAAATCCTCCAA
COII3p	TTAATCGATTTTTATTAGAAGGTCAAA	TTGCACCACAAATTTCAGAACA
ND3a	TCCCTTAAATTTTCTATTTATTGATGAGG	GGAATTAAACCACTAAAAATTAAGTTAGC
ND3b	TTATTTTACTTTTAGGATTATTTCACG	CCTTATCCTTATATAATTTATTTGCCTTT
ND5	GCTAAATCAGGTAATCCCATTCTT	TTTATTAGGTTGAGATGGTTTAGGTTT
Cytb-a	CCAATTCGAAAAACACACCCTA	TTCGAGGGACTTTACCTCGTT
Cytb-b	TCCTTTAGGAATCAATAGAAATTTAG	TTCGAGGGACTTTACCTCGTT
ND1	TCATATCGATAACGAGGTAAAGTCC	CCTGTCATTAGATTTATTTTGTCTTT
gap1	AGGGGGTTTACCTCCATTTA	AATAAATGCATGAGCTGTTACAATA
gap2	TTACCACCTGCAGAACATTCA	CGTAATGAAGGTAAAGCAATAAAAA
gap3	GCAAGTACTGGTCTCTTAAACCAT	AGATGGGTCAAAAATTGAAAAA
gap4	CAGCCAATATAATTGCAGGACA	TCTCGACAAATATCTCGTCATCA
gap5	ATTTGAAGCTGCTGCCTGAT	CGAGCAGAAGATTTAGGGTCA
gap6	GACCCTAAATCTTCTGCTCGAA	GAGAAGTTTATAGCGGTTATGGAA
gap7	AACCACTGAAGAAATTAATATAACAAA	CATGGTAAATATTATTCAGGAATTT
gap8	GCTCCCTCACAAACTGAGAAA	TTTCATTTGATGCAATTCTAGATACA
gap9	TTAGGCCCTTTACGAATTTGA	GCGACCTCGATGTTGGATTA
gap10	CAACATCGAGGTCGCAAAC	TTGACTGTACAAAGGTAGCATAAT
gap11	TTATGCTACCTTTGTACAGTCA	TGTATCTTGTGTATCAGAGTTTATT

The FASTA-formatted BUR38 mitochondrial sequence was imported into a local database using BioEdit [29], and was queried with *O. nubilalis* protein and tRNA sequences from GenBank ID: AF442957.1. Protein cds were defined based on invertebrate mitochondrial translation table using the Virtual Ribosome (http://www.cbs.dtu.dk/services/VirtualRibosome/) [30]. The tRNA gene boundaries and folded structures were defined using tRNAscan-SE (http://lowelab.ucsc.edu/tRNAscan-SE/) [31] with the following parameters: (i) search mode – organellar; (ii) search type – cove only; (iii) cove cut off score −15; (iv) translation code used – invertebrate mitochondrial; (v) maximum intron length – 40; and (vi) pseudogene checking – disabled. The mitochondrial sequences were imported into the Artemis visualization and annotation tool [32], positional information of sequence features was added, and the annotated data exported in GenBank(.gb) format.

### Phylogenetics

The FASTA-formatted mitochondrial genome assembly for *M. vitrata* was used in a query in the National Center for Biotechnology Information (NCBI) non-redundant nucleotide database (nr) with the blastn search algorithm [33]. The resulting hits to full mitochondrial genome sequences for species of Lepidoptera were downloaded in FASTA format. The FASTA formatted mitochondrial genome sequences were imported into the MEGA 4.0 software package sequence alignment application [34] and a multiple sequence alignment was performed with the ClustalW algorithm using default parameters (gap opening penalty 15, gap extension penalty 6.66, weight matrix IUB, and transition weight of 0.5). The nucleotide diversity (p), base composition, transition to transversion bias (R; [35]), disparity in substitution among nucleotide sites (Disparity Index), and test substitution homogeneity [36] of the aligned sequence was analyzed by MEGA 4.0 [34].

Maximum likelihood analysis of derived amino acid sequences, to infer the phylogenetic relationships among mitochondrial genome sequences, was performed using the PHYLIP software package [37]. Concatenated protein sequences from each species of Lepidoptera that has a complete mitochondrial genome within GenBank (current December 7, 2010) were imported into the MEGA 4.0 sequence alignment suite, aligned using the ClustalW algorithm (default parameters using the PAM protein weight matrix), and all gaps deleted manually. One thousand bootstrap pseudo-datasets were constructed using the program seqboot, which was used as input into ProML that used a Hidden Markov Model method to infer evolutionary rates among residue positions [38] with the Jones, Talyor, and Thorton [39] probability matrix. An unrooted majority rule consensus tree was generated using Consense, and viewed using TreeView 1.6.6 [40].

### 454 EST sequencing and mitochondrial transcript mapping

A total of 60 LPB larvae comprising of 3^rd^, 4^th^ and 5^th^ instars (20 each from Taiwan, Puerto Rico and Australia) were dissected to obtain the midgut and the salivary-gland tissues. Total RNA was extracted from these tissues using a TRIzol® Reagent protocol (Invitrogen, Carlsbad, CA) according to the manufacturer's instructions. RNA was quantified on a NanoDrop 2000 (Thermo Scientific, Wilmington, DE). First-strand cDNA was synthesized from 1 µg of total RNA using a BD Smart PCR cDNA synthesis kit (BD Biosciences, San Jose, CA). The first-strand cDNA was then amplified by a BD mix for 15 cycles (BD Biosciences, San Jose, CA) following the manufacturer's protocol except that a modified CDS II/3′ primer 5′ – TAG AGG CCG AGG CGG CCG ACA TGT TTT GTT TTT TTT TCT TTT TTT TTT VN -3′ (IDT Inc.) was used to avoid long homopolymer repeats. Subsequent to first-strand synthesis, the cDNA was then amplified using PCR Advantage II polymerase (Clontech Inc.) with the following thermal cycling program: (i) 1 min at 95°C, (ii) 21 cycles of 95°C for 7 sec, and 68°C for 6 min. A 2 µl aliquot of the PCR product was analyzed on a 1% agarose gel to determine the amplification efficiency. The PCR product was then subjected to SfiI digestion (10 units) for 2 h at 50°C to remove the concatemers formed by CDSIII/3′ and the SMART IV primers. A Qiaquick PCR purification kit (Qiagen, Valencia, CA) was used to remove the leftover primers and nucleotides from the amplified cDNA. The quality and quantity of the cDNA library was evaluated by both spectrophotometry and gel electrophoresis.

Sequencing and assembly: Amplified cDNA was submitted to the Keck Genomic Center (University of Illinois at Urbana Champaign) for library construction and sequencing. Two µg of amplified cDNA was used for library construction followed by pyro-sequencing on a Roche 454 GS-FLX (Roche, Basel, Switzerland) using established protocols [41]. The adaptor sequences were identified and the trim positions changed in .sff files using cross-match (http://www.phrap.org), sff tools from Roche (https://www.rocheapplied-science.com) and custom-built Java scripts. Sequences shorter than 50 nucleotides or containing homopolymers (in which 60% over the entire length of the read is represented by one nucleotide) were not included for assembly. The sequences were assembled with the Lasergene software (http://www.dnastar.com), using the modified .sff files. Raw sequence data were obtained from .sff files, and assembled into contigs using the Roche GS *De Novo* Assembler (*i.e.*, Newbler assembler), and data comprising all non-redundant contigs were exported to FASTA format.

A local database, MvEST01, containing all Newbler-assembled non-redundant contigs was constructed using BioEdit [29]. MvEST01 was queried with 13 *Ostrinia nubilalis* mitochondrial protein sequences and 21 tRNA sequences obtained from GenBank accession AF442957 using the tblastn search algorithms [33]. The results were then filtered for hits with ≥90% identity, and inclusive contigs were retrieved from MvEST1.

### Scaffolding of *M. vitrata* ESTs and transcript mapping

The EST reads were scaffolded against the *M. vitrata* mitochondrial genome assembly using the NextGENe software (Softgenetics®, State College, PA; parameters used were – matching base number > = 8 and matching base percent > = 70), and contiguous overlapping sequences exported as consensus contigs in FASTA format. The coding region(s) of contig sequences were annotated manually via blastx search of the *M. vitrata* mitochondrial gene model developed previously, and derived peptide sequence were predicted using the Virtual Ribosome v.1.1 [30].

## Results and Discussion

### Sequence and annotation of the *M. vitrata* mitochondrial genome

**Figure 1:**
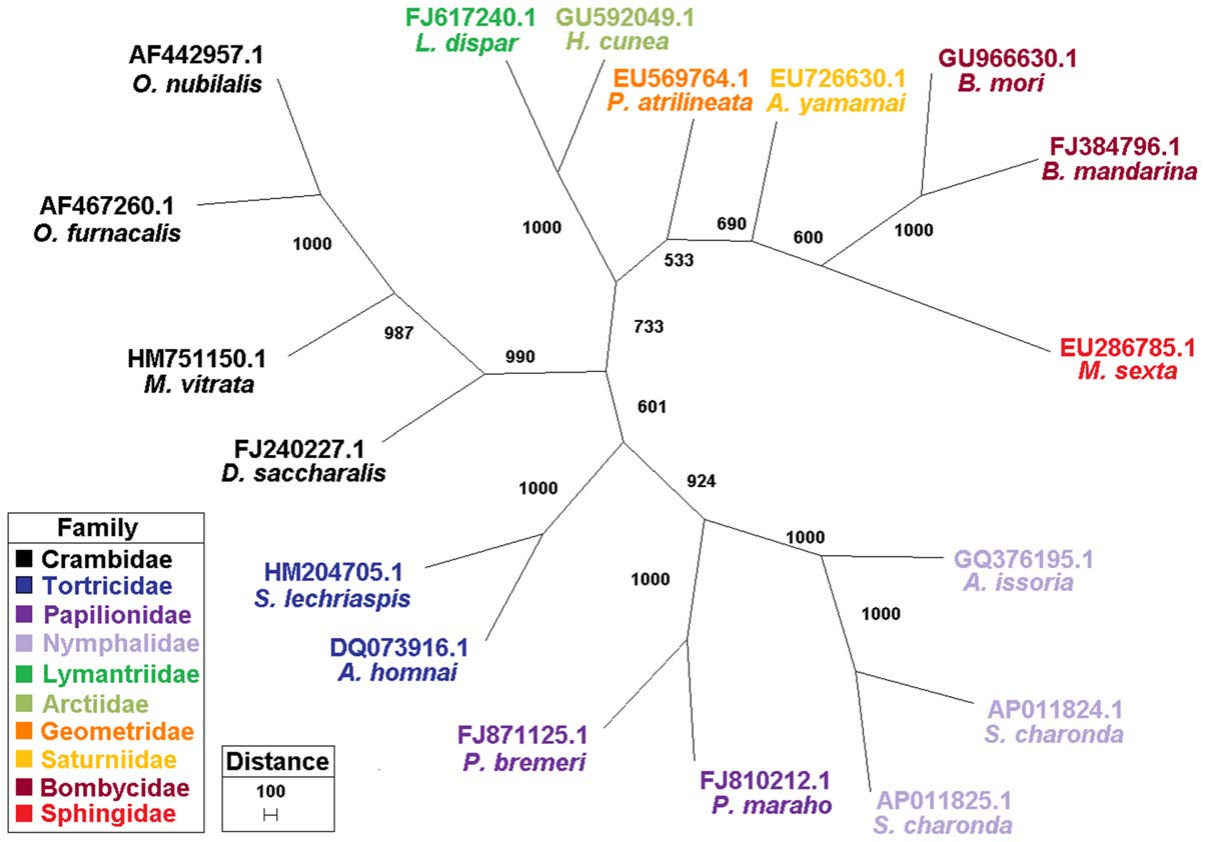
Phylogenetic relationships among mitochondrial genomes sequences. Phylogenetic relationships among complete mitochondrial genome sequences from the insect Order Lepidoptera using maximum likelihood estimations from a Hidden Markov Model, and branch support shown for 1000 bootstrap pseudoreplicates in a majority rule tree. The GenBank accession no., species, and Family assignment are indicated.

**Figure 2:**
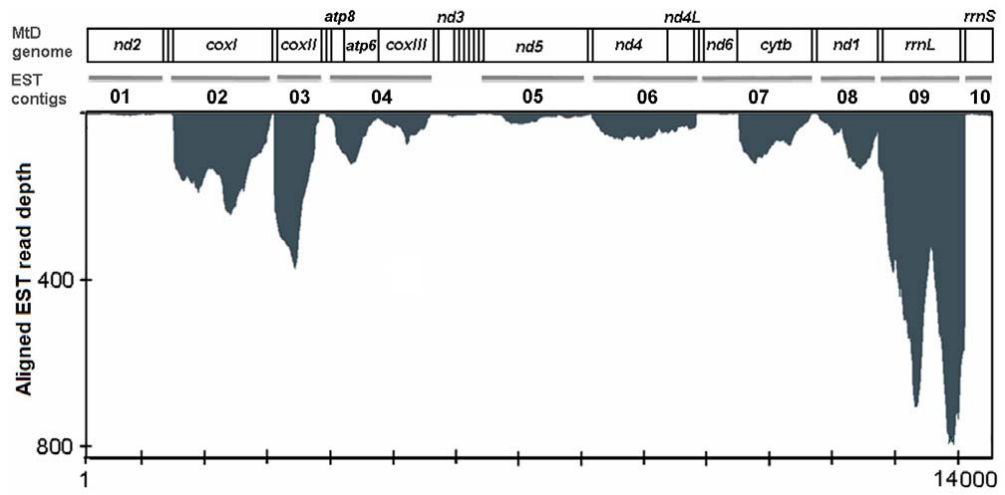
Transcript mapping to the mitochondrial genome. Transcript mapping of raw EST read data to respective positions on the *M. vitrata* mitochondrial genome (GenBank accession HZ751150), and assembled contigs representing polycistronic transcripts. The gene annotation and expression profile data for each of the contigs are presented in Table 4. Light strand encoded genes are indicated by asterisks (*), and arrows indicate the direction of transcription for polycistronic RNAs from the *M. vitrata* mitochondrial genome.

Nineteen overlapping PCR products generated using the oligonucleotide primer pairs shown in Table 1 yielded a total of 15,385 bp of sequence data. The data were assembled into a 14,054 bp partial sequence of the *M. vitrata* mitochondrial genome that was submitted to GenBank (HZ751150). This sequence lacks only the displacement loop (D-loop) that encodes the origin of replication or heavy strand promoter (HSP) and light strand promoter (LSP). The *M. vitrata* mitochondrial genome showed a high A+T nucleotide content of 80.0%, typical of animal and insect mitochondrial genome sequences [42], [43]. The A+T bias in *M. vitrata* is lower than that in any other mitochondrial genome sequence from Lepidoptera (Fig. 1), but this likely is the consequence of our partial sequence that does not contain the A-T rich D-loop. Although it does not include the entire mitochondrial genome, the *M. vitrata* assembly allowed for the identification and analysis of 2 rRNAs, 13 protein coding genes, and 19 of 22 tRNA genes (Fig. 2). The gene order and orientation seen in *M. vitrata* is typical for insect mitochondrial genomes (Table 2) [9], and is identical to that of the other Crambid species *O. nubilalis* and *O. furnacalis* [7]. Phylogenetic analyses indicated that the *M. vitrata* mitochondrial DNA grouped with a clade containing other species from the lepidopteran Family Crambidae, and clustered with other Families. A clear distinction between moth and butterfly species was apparent (Fig 1; alignment not shown). Thus, the use of the entire mitochondrial genome may clarify phylogenetic relationships that could not be determined using smaller sequence sampling methods [44].

**Table 2:** Summary of *Maruca vitrata* predicted tRNAs.

Gene	Strand	5′ start	3′ stop	Length (bp)	Start	Stop
*Nd*2	H	1	999	999	TTA	TAA
tRNA^TrpUGR^	H	1007	1074	68		
tRNA^Cys UGY^	H	1068	1131	64		
tRNA^TyrUAY^	L	1199	1137	61		
*cox*I	H	1207	2739	1533	CGA	TAA
tRNA^LeuUUR^	H	2735	2801	67		
*cox*II	H	2802	3486	685	ATG	T
tRNA^LysAAR^	H	3484	3554	71		
tRNA^AspGAY^	L	3557	3624	68		
*atp*8	H	3624	3785	162	ATT	TAA
*atp*6	H	3779	4453	675	ATG	TAA
*cox*III	H	4461	5249	789	ATG	TAA
tRNA^GlyGGN^	H	5252	5317	66		
*Nd*3	H	5318	5671	354	ATT	TAA
tRNA^AlaGCN^	H	5673	5738	66		
tRNA^ArgCGN^	H	5737	5802	66		
tRNA^AsnAAY^	H	5801	5866	66		
tRNA^SerAGN^	H	5869	5936	68		
tRNA^GluGAR^	H	5937	6001	65		
tRNA^PheUUY^	L	6069	6004	64		
*Nd* 5	L	7790	6260	1529	ATT	TAA
tRNA^HisCAY^	L	7872	7806	65		
*Nd* 4	L	9213	7872	1340	ATG	TAA
*Nd* 4L	L	9491	9213	277	ATG	TAA
tRNA^ThrACN^	H	9465	9532	68		
tRNA^ProCCN^	L	9591	9531	59		
*Nd* 6	H	9611	10040	430	ATT	TAA
*cyt*b	H	10377	11364	988		
tRNA^SerUCN^	H	11367	11432	66		
*Nd* 1	L	12378	11449	928	TTG	TAG
tRNA^LeuCUN^	L	12455	12388	66		
*rrn*L	L	13780	12476	1303		
tRNA^ValGUN^	L	13852	13804	47		
*rrn*S	L	13863	14139	277		

The tRNAs are presented in the order they occur on the mitogenome along with their beginning and ending nucleotide positions. The anticodon region detected and the corresponding amino acid the predicted tRNA transfer are presented. The orientation is indicated with respect to the heavy (H) or light (L) strand is indicated.

Protein coding regions encompassed 10,266 of the 14,052 bp assembled sequence (75.5%) and showed an A+T content of 82.9%, which is slightly higher than that of the entire sequence, but both were consistent with the high A+T content of insect [9], [43] and lepidopteran mitochondrial DNAs [45]. This AT-bias was reflected in the codon usage of the 14 PCGs (Table 3), where phenylalanine (F; UUU), isoleucine (I; AUU), leucine (L; UUA), methionine (M; AUA), and tyrosine (Y; UAU) were proportionately the most represented. The AT-bias in codon usage also was likewise observed in that 3218 of 3422 codons had third positions with either an A or T nucleotide (94.0%), and was significantly higher proportion than at the first codon positions (73%; χ^2^ = 4.69, *P*-value = 0.030) and second codon positions (69.8%; χ^2^ = 6.23, *P*-value = 0.013). This difference may reflect the selection for optimal codon usage [46] or codons that match the anti-codons of tRNAs [47].

**Table 3:** Codon usage in the *Maruca vitrata* mitochondrial genome showing a high A-T nucleotide bias in the 3rd (wobble) position (94.0%).

UUU(F)	315.0(1.82)	|	UCU(S)	98.0(2.69)	|	UAU(Y)	169.0(1.88)	|	UGU(C)	28.0(1.87)
UUC(F)	31.0(0.18)	|	UCC(S)	8.0(0.22)	|	UAC(Y)	11.0(0.12)	|	UGC(C)	2.0(0.13)
UUA(L)	426.0(5.26)	|	UCA(S)	12.0(2.06)	|	UAA(*)	10.0(1.82)	|	UGA(W)	88.0(1.96)
UUG(L)	9.0(0.11)	|	UCG(S)	1.0(0.05)	|	UAG(*)	1.0(0.18)	|	UGG(W)	2.0(0.04)
CUU(L)	26.0(0.32)	|	CCU(P)	74.0(2.47)	|	CAU(H)	60.0(1.82)	|	CGU(R)	12.0(0.94)
CUC(L)	2.0(0.02)	|	CCC(P)	8.0(0.27)	|	CAC(H)	6.0(0.18)	|	CGC(R)	0.0(0.00)
CUA(L)	23.0(0.28)	|	CCA(P)	38.0(1.27)	|	CAA(Q)	54.0(1.96)	|	CGA(R)	37.0(2.90)
CUG(L)	0.0(0.00)	|	CCG(P)	0.0(0.00)	|	CAG(Q)	1.0(0.04)	|	CGG(R)	2.0(0.16)
AUU(I)	394.0(1.91)	|	ACU(T)	83.0(2.35)	|	AAU(N)	202.0(1.87)	|	AGU(S)	31.0(0.85)
AUC(I)	18.0(0.09)	|	ACC(T)	8.0(0.23)	|	AAC(N)	14.0(0.13)	|	AGC(S)	1.0(0.03)
AUA(M)	240.0(1.85)	|	ACA(T)	49.0(1.39)	|	AAA(K)	73.0(1.70)	|	AGA(S)	76.0(2.09)
AUG(M)	19.0(0.15)	|	ACG(T)	1.0(0.03)	|	AAG(K)	13.0(0.30)	|	AGG(S)	0.0(0.00)
GUU(V)	71.0(2.07)	|	GCU(A)	75.0(2.52)	|	GAU(D)	55.0(1.75)	|	GGU(G)	57.0(1.19)
GUC(V)	4.0(0.12)	|	GCC(A)	9.0(0.30)	|	GAC(D)	8.0(0.25)	|	GGC(G)	0.0(0.00)
GUA(V)	59.0(1.72)	|	GCA(A)	35.0(1.18)	|	GAA(E)	68.0(1.89)	|	GGA(G)	117.0(2.45)
GUG(V)	3.0(0.09)	|	GCG(A)	0.0(0.00)	|	GAG(E)	4.0(0.11)	|	GGG(G)	17.0(0.36)

The methionine start codons, UTG, were used by 6 of 13 protein coding genes (46.2%). In contrast, an atypical Ile codon (AUU) was used to initiate protein synthesis in the *atp*8, *nd*3, *nd*5, and *nd*6 genes, and Phe (UUR) was used in the *nd*1 and *nd*2 genes, and Arg (CGA) was used for the *cox*I gene. The use of Ile for initiation of peptide synthesis has previously been reported in other insects [48] including Lepidoptera [6], [7], which suggests that it is common in this insect Order. Additionally, the *cox*1 gene was first annotated with an Arg (CGA) initiation codon for protein synthesis in *Drosophila yakuba* [9], and the same codon was also subsequently shown for the Lepidopteran species *B. mori* [6], *O. nubilalis* and *O. furnacalis* [7], *Adoxophyes honmai* [49], *Coreana rephaelis* [50], *Antheraea pernyi* [45], *B. mandarina* [51], *Ochrogaster lunifer* [52], *Artogeia melete* [53], *Eriogyna pyretorum* [54], and *Hyphantria cunea* [8]. The nucleotide sequence, TTAG, located immediately upstream of the putative Arg CGR start codon of *cox*I was proposed to serve in a non-standard initiation process [6]. Our alignment of the mitochondrial genome sequence from 19 species of Lepidoptera (Fig. 1; alignment not shown) indicated that this putative TTAG initiation was conserved among 15 of 27 (55.6%) *cox*I genes from full lepidopteran mitochondrial genomes (Fig. 1). The lack of absolute conservation of the TTAG suggests that there may be flexibility in the function of the sequence, or that it may not serve as an initiation codon. In any case, further study is required to determine the mechanism of *cox*I initiation, but transcript information provided by Stewart and Beckenback (2009) [55] and our data described below indicate that the Arg (CGR) indeed functions as the initiation codon.

The corresponding stop codons used by *M. vitrata* mitochondrial genes were predicted to be TAA in all instances except for *cox*II and ND1 where T and TAG motifs were observed, respectively (Table 2). Based on invertebrate mitochondrial genetics, we predict that the UAG codon serves as a stop codon, and it has only been observed in *M. vitrata*, *Ostrinia* sp. [7], *Parnassius bremeri* (GenBank ID: FJ871125.1), and *Parnassius maraho* (GenBank ID: FJ810212.1) mitochondrial genomes. The infrequent use of the TAG stop codon is likely a consequence of the high A+T bias at the third codon position [43]. The incomplete stop codon of *cox*II (T nucleotide only) was previously reported in insects [7], [9], and for other mitochondrial genes in bivalves [56] and mammalian species [3]. Mitochondrial RNA processing occurs by the tRNA punctuation model [3], wherein the cloverleaf-like secondary structures of tRNAs are required for the cleavage of polycistronic transcripts into mRNAs. Maturation of the mitochondrial mRNAs occur by the addition of poly(A) tails, and results in completion of the TAA stop codon for *cox*II (A nucleotides added via polyadenylation are underlined; [55]). Similarly, we present EST data in the following section that indicate that mature *M. vitrata cox*II transcripts are also polyadenylated.

### *M. vitrata* EST assembly and mitochondrial cistronic transcripts

The *de novo* assembly of the *M. vitrata* larval midgut and salivary EST read data resulted in a total of 3729 contigs (mean length 459.6±287.3 bp; range 96 to 3299 bp), of which 10 contigs were annotated as derived from the mitochondrial genome (1335.7±510.3 bp; Supplemental Data S1); these contigs resulted from the clustering of 7608 raw reads (Supplemental Data S2). The arrangement of genes within assembled contigs likely represent mature transcripts that are derived from tRNA punctuated cleavage of a polycistronic transcript [3], [57], and in *M. vitrata* it showed that three contigs had more than one gene (contig 4: *atp*8, *atp*6, and *cox*III; contig 6: *nd*4 and *nd*4L; and contig 7: *nd*6 and *cyt*b; Fig 2; Table 4). The *atp*6 and *atp*8 genes, as well as the *nd*4 and *nd*4L genes, were located upon the same mature mitochondrial transcript within the Dipteran insects *D. melanogaster* and *D. pseudoobscura* [55], [57], [58] and the swine *Sus scofa* [10]. According to the tRNA punctuated model, all *M. vitrata* mitochondrial polycistronic transcripts are cleaved at junctions with tRNAs, such that the 3′ end of cistrons are each interspersed by a tRNA gene [3], [5]. By default, mitochondrial genes on the same cistron that are contiguous within the genome lack an intervening tRNA. This contiguous structural arrangement of the *atp*8/*atp*6/*cox*III, *nd*4/*nd*4L, and *nd*6/*cyt*b genes was observed in the *M. vitrata* mitochondrial genome, and was also predicted within the resulting mature transcripts (cistrons; Fig. 2; Table 4).

**Table 4:** The quantitative expression profile of cistrons transcribed from *Maruca vitrata* mitochondrial genome.

Contig (cistron)	Genes within contig	Contig length	Total nt	Mean depth (nt)	Read no.	Proportion
MvMtD_01	*nd*2	1,033	7,829	7.58	24	0.2
MvMtD_02	*cox*I	1,532	265,789	290.16	916	6.9
MvMtD_03	*cox*II	713	239,189	300.49	796	6.0
MvMtD_04	*atp*8, *atp*6, *cox*III	2,049	171,490	83.69	558	4.2
MvMtD_05	*nd*5	1,736	27,891	16.07	81	0.6
MvMtD_06	*nd*4	1,640	89,043	54.29	297	2.2
MvMtD_07	*nd*6, *cyt*b	1,768	117,208	66.29	359	2.7
MvMtD_08	*nd*1	1,022	86,645	84.48	278	2.1
MvMtD_09	*rrn*L (16s rDNA)	1,408	1,053,548	748.29	4,288	32.1
MvMtD_10	*rrn*S (12s rDNA)	456	2870	6.29	11	0.1

The number of raw reads (Read no.) that were scaffolded to each contig (cistron) was used to calculate the relative proportion of mitochondrial transcripts within the pooled M. vitrata midgut and salivary gland EST library.

The overlap of the gene coding sequence between those of *M. vitrata atp*8 and *atp*6 (7 bp), and *nd*4 and *nd*4L (1 bp), would logically dictate that the mature transcripts remain intact in order to preserve their respective coding sequences. This scenario was also predicted due to the absence of an intervening tRNA that would be necessary for transcript cleavage [3], [5]. To our knowledge, the polycistronic *atp*8/*atp*6/*cox*III transcript identified from *M. vitrata* has yet to be reported in insects despite ancestral genome arrangements that likely would lead to this tricistronic transcript. *Drosophila melanogaster* showed polyadenylation at the 3′ terminus of bicistronic *atp*8/*atp*6 and monocistronic *cox*III mitochondrial transcripts [55], which suggests that variation in mitochondrial transcript cleavage events may exist among insect species. Indeed, differences occur between species of salamander, where a tricistronic *atp*8/*atp*6/*cox*III transcript from *Ambystoma tigrinum* is processed into *atp*8/*atp*6 and coxIII transcripts within *A. mexicanum* [59]. Our mitochondrial transcript mapping data are the first to be reported for a lepidopteran, so direct comparison with data from other species cannot readily be made. Undoubtedly the inclusion of mitochondrial transcript mapping data from additional species will allow us to understand the functional variation in mitochondrial transcript processing.

Only two out of the eight (25%) mature processed *M. vitrata* mitochondrial transcripts have possible poly(A) tails (coxII and atp8/atp6/coxIII cistrons; Supplemental Data S1), which is in contrast to the observation of 3′ polyadenlyation for all cistronic transcripts in *Drosophila* [55]. Generation of initial *M. vitrata* cDNA by use of a poly(T) oligonucleotide would indicate that adenlyated forms of each cistron would be within the EST assemblies, if indeed present within the biological samples. The post-transcriptional 3′ addition of adenosine nucleotides results in increased transcript stability in many systems, but it has been linked to increased degradation in plant mitochondria [60]. RNAi knockdown of the enzymatic machinery involved in polyadenylation was previously reported to produce no apparent change in mitochondrial transcript stability in humans [61], [62]. Hence, the role of adenylation in mitochondrial transcript stability remains unknown [63]. Adenylation is required by the *cox*II transcript for the completion of its termination codon, but the functional or protective role of poly(A) tails remains uncertain given the absence from most cistronic transcripts in *M. vitrata*.

### Scaffolding of *M. vitrata* ESTs and transcript mapping

Since polycistronic transcripts are cleaved to generate mRNAs for each mitochondrial gene-derived cistron, the initial molar ratios are equal and subsequent variation in processed transcript quantities result from differences in mRNA stability [64]. The scaffolds comprised of short read EST data from *M. vitrata* were representative of full-length transcripts (Fig. 2), and appear not to be significantly influenced by reported over-representations of the 3′ end of fragments sometimes observed from 454 data [57]. Furthermore, the scaffolds aligned with our previous contig (cistron) assemblies that were representative of mature processed mitochondrial transcripts, and showed a read depth of 1 to 784 (Fig. 2; Table 4). Similar data from Next Generation Sequencing (NGS) technologies has been used to characterize gene expression [57], and applied to describe the sequence and relative quantities of processed mitochondrial-derived transcripts [10], [11]. The quantification described in those studies used simple calculations of the proportion of raw sequence reads mapped to each transcript (cistron) as a relative measure of cellular transcript level, which was used as an estimation of gene expression [57], [65].

A similar quantification of *M. vitrata* mitochondrial-derived transcript levels from scaffolded EST data indicated a ∼321-fold variance in cistron level, where the *rrn*L gene was present in the highest proportion (Table 4). The *rrn*L transcript was also present in the highest proportion of *D. melanogaster* [11] and porcine cistrons [10], and the analogous large abundance of rrnL transcripts in human expression data was attributed to the presence of a second HSP upstream of the gene that terminates in proximity near the tRNA^LeuUUR^ downstream of the 16 S rRNA [66], [67], [68]. The HSP and LSP sequences have diverged rapidly in mammalian systems [69], such that defining these functional regions is difficult by comparative sequence analyses [70]. The proportional range of cistron levels for those that encode for proteins involved in the same process showed ≤11.15-fold difference in cistron quantities for the NADH dehydrogenase complex (*nd*1 vs. *nd*2), and a ≤3.59-fold variance among cistrons for the cytochrome *c* oxidase (*cox*II vs. *cox*III). The estimated expression levels of *cox*I and *cox*II were approximately equal in *M. vitrata* ESTs. An overall lack of correlation between transcript levels within a protein complex has been observed among species of *Drosophila* [11], and likely can be attributed to differences in individual transcript stabilities [64]. Furthermore, these estimates do not take into account any differences in protein stability or turnover in the cell that would influence cellular metabolic processes [71]. The expression data represent additional data for annotation, but comparison with ESTs derived from different *M. vitrata* tissues or growth stages are likely to provide valuable information regarding metabolic variation.

### Conclusions

This is the first study of a species of Lepidoptera to provide functional annotation of a complete mitochondrial genome using gene expression data. These results will be valuable for future studies to provide comparisons for other species of Lepidoptera and to describe tissue-specific mitochondrial transcript processing pathways. This study provides a paradigm for the mating of EST and functional mitochondrial genome annotations that can be accomplished for any species for which both data sets are present.

## Supporting Information

Data S1 *Maruca vitrata* mitochondrial DNA assembled contigs.
(TXT)

Data S2 *Maruca vitrata* 454 raw reads corresponding to the mitochondrial genome assembly.
(TXT)
